# Underlying mechanism of the cyclic migrating motor complex in *Suncus murinus*: a change in gastrointestinal pH is the key regulator

**DOI:** 10.14814/phy2.13105

**Published:** 2017-01-13

**Authors:** Anupom Mondal, Kouhei Koyama, Takashi Mikami, Taichi Horita, Shota Takemi, Sachiko Tsuda, Ichiro Sakata, Takafumi Sakai

**Affiliations:** ^1^ Department of Life Nano‐BioStrategic Research DivisionGraduate School of Science and EngineeringSaitama UniversitySaitamaJapan; ^2^ Area of Regulatory BiologyDivision of Life ScienceGraduate School of Science and EngineeringSaitama UniversitySaitamaJapan

**Keywords:** Gastrointestinal pH, MMC, motilin

## Abstract

In the fasted gastrointestinal (GI) tract, a characteristic cyclical rhythmic migrating motor complex (MMC) occurs in an ultradian rhythm, at 90–120 min time intervals, in many species. However, the underlying mechanism directing this ultradian rhythmic MMC pattern is yet to be completely elucidated. Therefore, this study aimed to identify the possible causes or factors that involve in the occurrence of the fasting gastric contractions by using *Suncus murinus* a small model animal featuring almost the same rhythmic MMC as that found in humans and dogs. We observed that either intraduodenal infusion of saline at pH 8 evoked the strong gastric contraction or continuously lowering duodenal pH to 3‐evoked gastric phase II‐like and phase III‐like contractions, and both strong contractions were essentially abolished by the intravenous administration of MA 2029 (motilin receptor antagonist) and D‐Lys3‐GHRP6 (ghrelin receptor antagonist) in a vagus‐independent manner. Moreover, we observed that the prostaglandin E2‐alpha (PGE2_‐_ *α*) and serotonin type 4 (5HT4) receptors play important roles as intermediate molecules in changes in GI pH and motilin release. These results suggest a clear insight mechanism that change in the duodenal pH to alkaline condition is an essential factor for stimulating the endogenous release of motilin and governs the fasting MMC in a vagus‐independent manner. Finally, we believe that the changes in duodenal pH triggered by flowing gastric acid and the release of duodenal bicarbonate through the involvement of PGE2_‐_ *α* and 5HT4 receptor are the key events in the occurrence of the MMC.

## Introduction

The term migrating motor complex (MMC) was first defined by Szurszewski (1969) as the result of the recording of a cyclic pattern of intestinal migrating myoelectrical activity in dogs. During the interdigestive state, the stomach and small intestine undergo a temporally coordinated cyclic motor pattern in many monogastric animals (including rodents, dogs, pigs, rabbits, and humans), known as the migrating motor complex (MMC) (Szurszewski 1969; Vantrappen et al. 1977). The MMC is composed mainly of three typical phases: I, a period of motor quiescence with no contractions; II, a period characterized by intermittent, preceding irregular and low‐amplitude contractions; and III, a period characterized by clustered, regular, and high‐amplitude contractions (Itoh 1997). The MMC is generally accepted as “the housekeeper of the gastrointestinal tract”. In particular, the phase III contractions of the MMC have been considered to have physiological significance in the cleansing of secretions, debris, and microbes during fasting, to enable the stomach to be prepared to receive the next meal (Vantrappen et al. 1977). In contrast, impaired gastric phase III contraction may result in prolonged retention of gastric contents as well as bacterial overgrowth, which may lead to various symptoms (Vantrappen et al. 1977). However, the underlying pathway controlling the MMC remains incompletely elucidated. Moreover, it has been observed that the occurrence of the MMC is regulated in an ultradian rhythmic period of 90–120 min intervals in the gastrointestinal (GI) tract in humans and dogs (Itoh et al. 1976; Vantrappen et al. 1979a). However, whether the occurrence of such ultradian rhythmic patterns of the MMC is found in multiple species during the interdigestive state with a stipulated time interval is a question that remains to be answered.

The MMC has been found to be a complex system and may be regulated by a composite of several gut peptides, the enteric nervous system, and/or the vagus nerve (Deloose et al. 2012). It has been well documented that motilin, a 22‐amino acid peptide first isolated from porcine intestinal mucosa (Brown et al. 1973), plays an important role in regulating the phase III contractions of the MMC in humans and dogs. Cyclic changes in plasma motilin levels were found to strongly correlate with the appearance of gastric phase III in humans (Vantrappen et al. 1979a; Janssens et al. 1983) and dogs (Itoh et al. 1976, 1978; Hall et al. 1983). Moreover, it has been observed that intravenous (IV) administration of motilin induces gastric phase III‐like contractions in humans (Janssens et al. 1983) and dogs (Itoh et al. 1976; Wingate et al. 1976). Ghrelin, another polypeptide with a structure resembling that of motilin (Kojima et al. 1999), is also associated with the MMC in rats (Masuda et al. 2000) and mice (Zheng et al. 2009). However, the involvement of ghrelin in GI contractions is controversial and has been found to be species‐specific (Masuda et al. 2000; Ohno et al. 2006; Tack et al. 2006; Zheng et al. 2009). A recent human study clearly showed that plasma motilin, but not ghrelin, is associated with the MMC; however, it also suggested that a certain amount of ghrelin may necessary to initiate motilin‐induced gastric contraction (Deloose et al. 2015). Moreover, it was recently observed that a low amount of ghrelin is necessary for motilin‐induced gastric contraction in *Suncus murinus (S. murinus)* (Kuroda et al. 2015). Thus, under physiological conditions, it appears that the cyclic release of motilin may be the main regulator controlling the occurrence of gastric phase III of the MMC in an ultradian time period. Nevertheless, since the discovery of motilin in the 1970s, comparatively little progress has been made in elucidating the precise mechanism of motilin release.

Several possible factors have been suggested to be involved in the release of motilin from the duodenum and jejunum that regulates the gastric MMC. For example, luminal serotonin (5HT), a biogenic amine that regulates GI motility, stimulates duodenal contractions resulting in increased luminal pressure to initiate motilin release (Takahashi 2012). However, progress in describing the comprehensive mechanism remains slow, in part because of the lack of a suitable animal model comparable to humans and dogs. Due to motilin and motilin receptor pseudogenes (He et al. 2010), mice and rats are not suitable candidates for an animal model for humans. Moreover, the characteristic features of the rodent gastric MMC are different, i.e., the rat shows a shorter interval MMC cycle (<20 min) (Fujino et al. 2003; Tatewaki et al. 2003; Ariga et al. 2007) compared to that of humans and dogs.

To address this question, we focused on the house musk shrew (*Suncus murinus;* order: Insectivora, referred to as suncus in a laboratory context), which has been considered a suitable model animal for GI motility studies. The general appearance of the gastric mucosa of suncus is similar to that of human and dogs, and differs from that of some other widely used experimental small animals, such as hamsters, rats, and mice (Kanamori et al. 1989). Unlike in rats and mice, the complementary DNA sequences of motilin and ghrelin in *S. murinus* have already been identified using polymerase chain reaction cloning (Ishida et al. 2009; Tsutsui et al. 2009). Furthermore, the motilin receptor (G protein‐coupled receptor 38; GPR38) and growth hormone secretagogue receptor (GHSR) genes in *S. murinus* have already been sequenced (Suzuki et al. 2012). Moreover, the freely moving fasted GI tract of suncus has demonstrated almost identical phase I, II, and III contractions of the MMC with a similar time interval (90–120 min) to those observed in humans and dogs (Sakahara et al. 2010; Mondal et al. 2012). In addition, it has been reported that the administration of motilin‐induced phase III‐like contractions in a dose‐dependent manner in vivo and in vitro (Sakahara et al. 2010; Mondal et al. 2011), while treatment with motilin antagonist eliminated the occurrence of spontaneous gastric phase III contractions (Mondal et al. 2012). Furthermore, it has been shown that motilin‐induced suncus gastric contractions were mediated through the myenteric plexus in a vagus‐independent manner (Mondal et al. 2011). These results indicated that *S. murinus* is a suitable small model animal showing characteristic features and mechanisms of the gastric MMC cycle.

In the present study, we used anesthetized suncus and studied the mechanism of the MMC by controlling duodenal changes in pH and the release of 5HT through the involvement of specific receptor subtypes. Moreover, to verify the results in the anesthetized animals, conscious animals were also used to elucidate the factors underlying the occurrence of the MMC in 90–120 min intervals in a fasted condition.

## Methods

### Ethical approval of the study protocol

All procedures were approved by and performed in accordance with the guidelines of the Committee on Animal Research of Saitama University (Saitama, Japan). All efforts were made to minimize animal suffering and to reduce the number of animals used in the experiments.

### Animals

Experiments were performed using adult male (10–20 weeks of age) and female (5–20 weeks of age) suncus of an outbred KAT strain established from a wild population in Kathmandu, Nepal (Oda et al. 1992), weighing between 50 and 100 g. Animals were housed individually in plastic cages equipped with an empty can for a nest box under controlled conditions (23 ± 2°C, lights on from 8:00 to 20:00) with free access to water and commercial feeding pellets (number 5P; Nippon Formula Feed Manufacturing, Yokohama, Japan). The metabolizable energy content of the pellets was 344 kcal/100 g. The pellets consisted of 54.1% protein, 30.1% carbohydrates, and 15.8% fat.

### Animal surgery for GI motility recording in vivo

After fasting for 3–5 h, each animal was anesthetized with an intraperitoneal (IP) injection of the combination of midazolam (4 mg/kg; psychotropic; Sandoz K.K., Yamagata, Japan), Domitor (0.3 mg/kg; pain relief and suppression; Nippon Zenyaku Kogyo Co., Fukushima, Japan), and Vetorphale (5 mg/kg; pain relief after operation; Meiji Seika Pharma Co., Tokyo, Japan). The methodology of animal surgery, implantation of transducer, and recording of GI motility were followed as described previously (Mondal et al. 2012; Miyano et al. 2013; Kuroda et al. 2015; Yoshimura et al. 2016). In brief, through a midline laparotomy, a strain gauge force transducer developed in our laboratory (Mondal et al. 2012; Miyano et al. 2013; Kuroda et al. 2015; Yoshimura et al. 2016) was sutured to the dorsal portion of the lower gastric body and on the serosal surface of the duodenal body (3 cm distal to the antro‐pyloric junction) for measuring the circular muscle contractions. Waterproofing and response properties were checked in all transducers before implantation. A silicon‐coated wire (RSF 66/0.03; Sanyo Electric Wire, Osaka, Japan) from the transducer was exteriorized through the abdominal wall and passed under the skin toward the back of the middle neck.

For the IV infusion of either drugs or a test compound, a silicone tube (1.0 mm OD × 0.5 mm ID; Kaneka Medics, Osaka, Japan) was inserted into the right jugular vein and exteriorized to the back of the neck on the right side. The catheter was filled with heparinized saline (100 units/mL) to prevent coagulation. For intraduodenal (ID) infusion, a small stab wound (2–3 mm) was made in the wall of the duodenum (5 mm distal to the pylorus). A silicone tube (1.0 mm OD × 0.5 mm ID) was then inserted and fixed in place with a purse‐string suture. The tail end of the silicone tube was passed subcutaneously to the back of the neck on the left side.

In the vagotomy experiments, a truncal vagotomy was performed in suncus subjected to both duodenal cannulation and jugular vein catheterization. The stomach and lower esophagus were exposed, and the dorsal and ventral vagus nerves were isolated. Both branches of the vagus nerves were cut, and all the neural connections in the resected area were peeled away completely using wiping tissues (Kimwipes; Nippon Paper Crecia, Tokyo, Japan). The vagus nerves were exposed only without any cuts or peeling in the sham‐operated suncus. All suncus were allowed to recover from the surgery for at least 2 days before the day of the experiment.

### Recording gastric motility in anesthetized and conscious states

GI motility recording under various conditions was performed on the third day after surgery. To record the GI motility under anesthetized conditions, an IP injection of 15% urethane solution at a dose of 1 mL/100 g body weight (BW) was administered after an overnight fasting. On the other hand, interdigestive gastric motility was measured in conscious, freely moving suncus. To record the GI motility, wires from the transducer were connected to an amplifier; the amplified signals were then converted by an analog‐digital converter (ADC‐20/24, Pico Technology Ltd., St Neots, UK). The digital signals were then recorded by PicoLog software (Pico Technology Ltd.) with a sampling interval of 100 msec. The recorded signals were processed with a frequency cutoff of 10 Hz using Chart 5 Reader software (ADInstruments, Ltd., Dunedin, New Zealand). It is noted that, under anesthetized condition, animals did not show any physiological phase II and III activity. Therefore, this condition allowed us to observe the clearer effect of the ID saline solution with different pH and the agonist or antagonistic activity on the gastric contractions. Thus, before ID or IV infusion of each drug or solution, the gastric or duodenal contractile pattern was remained as phase I‐like showing no irregular or strong contractions. Therefore, the obtained gastric and duodenal contraction in each study was quantified by comparing with the positive control motilin (300 ng/kg)‐induced gastric contractions. Gastric motility was quantified by calculating the motility index (MI; %) as the percentage of the area under the curve (AUC) of the control motilin (300 ng/kg)‐induced gastric contractions in the anesthetized animal, which was equivalent to the integrated area between the contractile wave and baseline of the adjacent control contraction.

### Drugs used

Synthesized suncus motilin (Scrum Inc., Tokyo, Japan), D‐lys3‐GHRP6 (ghrelin receptor antagonist, DLS; Bachem, Torrance, CA) (Mondal et al. 2012; Kuroda et al. 2015), and GR 125487 (5HT4 receptor antagonist, GR; Tocris Bioscience, Ellisville, MO) (Nakajima et al. 2010) were dissolved in 0.1% bovine serum albumin/phosphate‐buffered saline (BSA/PBS). MA‐2029 (motilin receptor antagonist, MA; kindly donated by Chugai Pharmaceutical Company, Tokyo, Japan) (Mondal et al. 2012; Kuroda et al. 2015) was dissolved in HCl‐saline (80 *μ*l 1N HCl in 50 mL saline). Ondansetron (5HT3 receptor antagonist, OND; Hikari Pharmaceutical, Imado, Japan) (Nakajima et al. 2010) and BIMU8 (5HT4 receptor agonist, Santa Cruz Biotechnology, Inc., Santa Cruz, CA) were dissolved in distilled water. Indomethacin (Indo; Sigma‐Aldrich Co., St. Louis, MO) (Thor et al. 1985; Lichtenberger et al. 2015) was dissolved in 1 eq ethyl alcohol (final concentration: 0.01% v/v). The independent administration of vehicles of each drug did not induce any effect (data not shown). All reagents were freshly prepared immediately before each experiment according to the manufacturer's instructions. Elsewhere the specificities and the preventive effect of the specific agonistic effect by the selective receptor antagonists have been checked for motilin (Sudo et al. 2008), ghrelin (Depoortere et al. 2003), 5HT4 receptor (Candura et al. 1996), and prostaglandin E2 (Takeuchi et al. 1986).

### Effect of ID infusions of alkaline and acidic saline on gastric contractions and analysis of MI

In the anesthetized suncus, bolus IV injection of motilin (300 ng/kg BW) evoked strong gastric contractions and was considered a positive control. Saline with different pH values (pH 6, 7, and 8, respectively) at a rate of 0.5, 1, and 2 mL/min for 1 min was infused in the presence and absence of either MA or DLS administration. IV infusion of MA (1 mg/kg/h, for 1 h) or DLS (6 mg/kg/h, for 1 h) was initiated 20 min before the ID infusion of saline with different pH values. The MI for the 20‐min period after the ID alkaline saline‐induced contraction with or without the MA and DLS treatment was compared to the MI for the adjacent 20‐min period control motilin‐induced gastric contraction. In the vagotomized animals, a similar process was followed to analyze the MI.

On the other hand, ID infusion of pH 3 saline (at a rate of 0.1 mL/min) was continued for 30 min. IV administration of MA (1 mg/kg/h) or DLS (6 mg/kg/h) was begun 20 min before the ID infusion of pH 3 saline, and continued for 90 min. In contrast, IV administration of GR (80 *μ*g/kg/h, for 70 min) or OND (100 *μ*g/kg/h, for 70 min) was initiated 10 min before ID infusion of pH 3 saline. To calculate the MI of the irregular contractions, first the time interval (min) from the point of ID administration of pH 3 saline to the starting point of strong gastric contractions was measured. Similarly, the time interval (min) of strong gastric contractions was calculated. Then, the AUC per min was calculated by dividing by the estimated interval time of irregular and strong gastric contractions, respectively. Finally, the MI was analyzed with the AUC/min with the respective AUC/min of the control motilin (300 ng/kg)‐induced gastric contraction in the anesthetized condition. The same time interval (min) was considered to calculate the AUC/min of both irregular and strong contractions under vehicle, MA, DLS, OND, and GR treatment.

### Effect on gastric contraction of IV infusions of 5HT under MA and DLS treatment and calculation of MI

To observe the effect of 5HT on gastric contractions, a bolus IV dose of 5HT (10 *μ*g/kg BW) was administered. MA (1 mg/kg/h) or DLS (6 mg/kg/h) was continuously IV infused for 30 min beginning 10–20 min after the administration of 5HT. The MI of the 5HT‐induced phase II‐ and III‐like gastric contractions was defined as the AUC/min. The MI during a 30‐min administration of antagonist was also calculated by the percentage of the AUC/min with control motilin‐induced contraction.

### Administration of 5HT4 receptor agonist and antagonist in the anesthetized and freely moving fasted conscious animals and measurement of MI

Following observation of control motilin (300 ng/kg)‐induced contractions, a selective 5HT4 receptor agonist, BIMU8, was administered by bolus IV injection (1 mg/kg) in the anesthetized animals. Either MA (1 mg/kg/h) or DLS (6 mg/kg/h) was continuously IV infused for 90 min starting 20 min before the administration of BIMU8. As previously described, the MI of the BIMU8‐induced irregular and strong gastric contractions was calculated using the AUC/min with the adjacent positive control contraction in the absence or presence of antagonists.

The effects of the 5HT4 receptor agonist (BIMU8) and the selective 5HT4 receptor antagonist (GR) on gastric motility in the freely moving fasted MMC were examined. Spontaneous gastric motility was recorded for 8–10 h in the fasting state. The definition of phase‐III contractions of the MMC in the conscious suncus was based upon that in dogs and humans: clustered contractions with amplitude of >8 g and lasting >5 min. Similarly, phase I was recognized as a period of motor quiescence and phase II was recognized as a period preceding irregular contractions. At 10 min after the completion of the spontaneous occurrence of phase III contractions, a bolus dose of BIMU8 (1 mg/kg) was injected IV. The duration (min) of the adjacent spontaneous gastric phase II and III contractions and BIMU8‐induced irregular (phase II‐like) and strong (phase III‐like) contractions were calculated.

Administration of GR (80 *μ*g/kg/h) was initiated at 50–70% (latter half of phase I) of the duration of the recent phase I and continued for 120 min. As a vehicle, the same volume of 0.1% BSA/PBS was administered.

### 5HT4 receptor agonist infusion in the vagotomized and sham‐operated anesthetized animals to observe the gastroduodenal contraction and MI measurement

The effect of BIMU8 (1 mg/kg) on gastroduodenal contractions was also examined and compared between the sham‐operated and vagotomized animals. As mentioned earlier, the MI of the BIMU8‐induced phase II‐ and III‐like gastric contractions was calculated in the presence of either vehicle or MA. In the duodenum, the motilin‐induced gastric contractions migrated to the duodenum, and were considered as an internal control of the duodenal contractions in the anesthetized suncus. The AUC/min was measured for the control migrated contractions. Similar to the gastric contractions, the duration (min) of the BIMU8‐induced duodenal clustered contractions (phase II‐like) and the duodenal strong propagated phase III‐like contractions were observed. Then the AUC/min of each contraction was measured by dividing by the estimated time. Finally, the MI was defined by the percentage of the AUC/min to the AUC/min of the motilin‐induced duodenal propagated contraction (internal positive control) in the presence of vehicle or MA.

### Administration of indomethacin and measurement of MI

Infusion of indomethacin (Indo; 10 mg/kg/h) or vehicle was initiated 10 min before the ID infusion of pH 3 saline, and continued for 70 min. Gastric phase II‐ and III‐like contractions induced by ID pH 3 saline infusion were quantified by MI of the percentage of the AUC/min in the presence of either vehicle or Indo. To observe the effect of Indo on the BIMU8‐induced gastroduodenal contractions, IV infusion of a dose of 10 mg/kg/h of Indo was initiated 20 min before the bolus administration of BIMU8 (1 mg/kg). Calculation of the MI of the BIMU8‐induced gastroduodenal motility in the presence of vehicle and Indo was performed as described in the previous section.

### Statistical analysis

We performed at least three individual experiments to record the data. The results of the experiments are expressed as the mean ± standard error of the mean (SEM). The numbers of animals are represented as “N” in the figure legends. We used GraphPad Prism 5 software (GraphPad Software Inc., La Jolla, CA) to analyze the data. One‐way analysis of variance (ANOVA) followed by Tukey's multiple comparison tests and Student's *t*‐test were used for statistical analysis of data. A value of *P* < 0.05 was considered statistically significant.

## Results

### The effect of ID infusion of alkaline pH saline on gastric contractions

**Figure 1:**
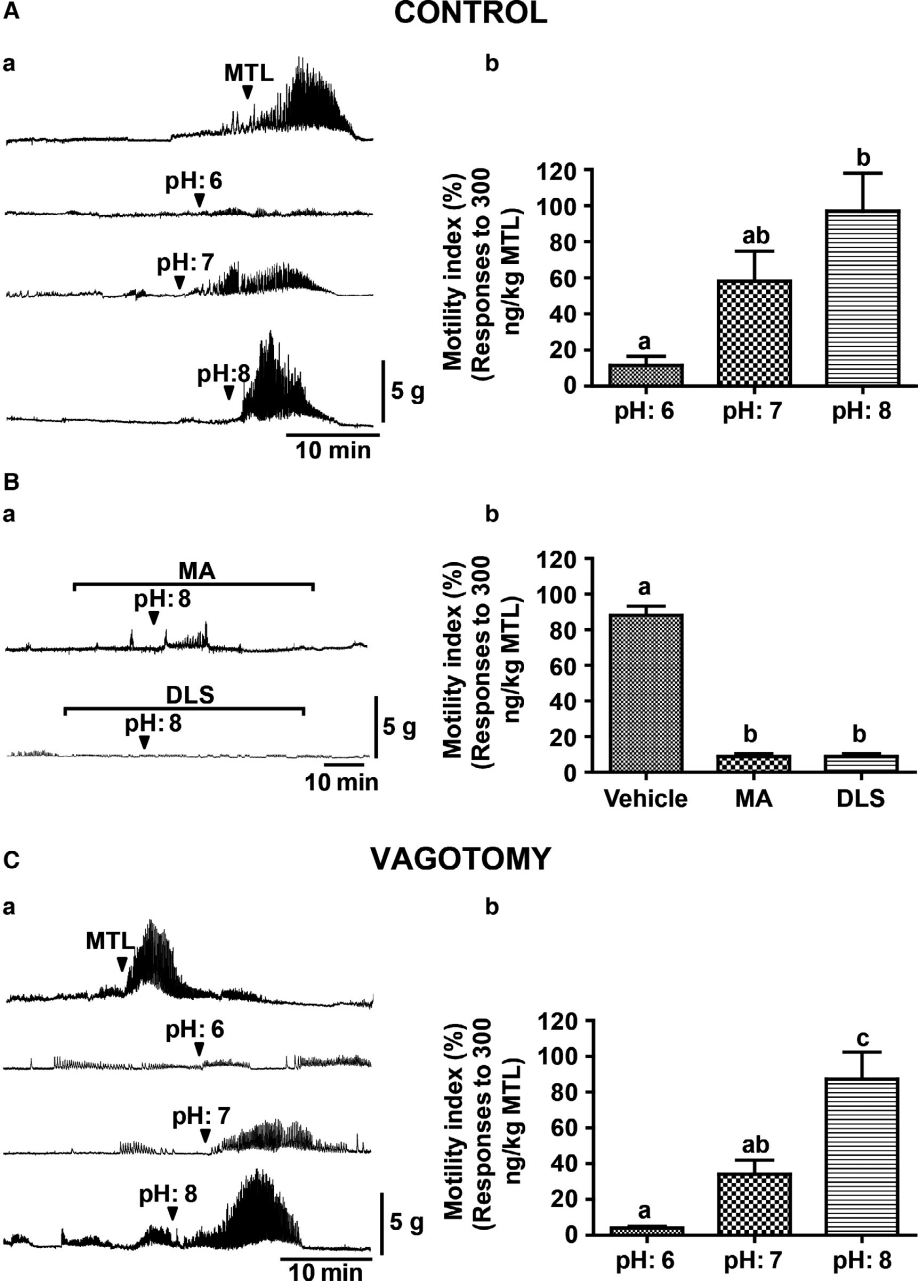
Effect of intraduodenal (ID) infusion of saline with different pH values on gastric contractions in the anesthetized animal. (Aa) Representative traces showing evoked gastric contractions by IV bolus injection of motilin (MTL, 300 ng/kg body weight [BW]) and ID injection of saline (2 mL/min, for 1 min) with pH 6, pH 7, and pH 8 respectively. (Ab) Motility index (MI) of gastric contractions caused by saline with different pH values. ID infusion of pH 8 saline induced maximum contractions. (B) Effect of MA and DLS treatment on duodenal alkaline (pH 8)‐induced gastric contraction (a). MI values in the antagonist‐treated group were significantly lower than those for the vehicle; however, MIs between the antagonist‐treated groups did not significantly differ (b). (Ca) Traces showing the gastric contractions induced by the MTL (300 ng/kg) as control and ID infusion of pH 6, 7, and 8 saline, in vagotomized animals. (Cb) Calculated MI showed similar strong gastric contractions with ID infusion of pH 8 saline. Each histogram represents the mean ± standard error of the mean (SEM), *N* = 3; Repeated measures analysis of variance (ANOVA) followed by Tukey's multiple comparison test; Different letters denote significant (*P* < 0.05) differences. Arrowheads indicate the timing of the administration of reagents.

Motilin at a dose of 300 ng/kg has been shown to induce phase III‐like contractions in the fasted conscious suncus (Sakahara et al. 2010; Miyano et al. 2013; Kuroda et al. 2015). Similarly, in the present study with anesthetized suncus, motilin (300 ng/kg) also induced gastric phase III‐like contractions, and these contractions were considered internal control contractions (Fig. 1Aa). We examined whether ID changes in pH could affect gastric contractions. We observed that ID infusion of saline at different pH values (pH 6, 7, and 8) at a rate of 2.0 mL/min for 1 min induced gastric contractions in a pH‐dependent manner (Fig. 1Aa), and that the MI of the ID infusion of pH 8 saline with a volume of 2 mL was the maximum, and similar to motilin‐induced phase III‐like contractions (Fig. 1Ab).

To determine the involvement of motilin and ghrelin in the gastric contractions induced by ID infusion of pH 8 saline, we pretreated with the motilin receptor antagonist, MA‐2029 (MA), or ghrelin receptor antagonist, D‐lys3‐GHRP6 (DLS), by IV administration. In the presence of either MA or DLS, the ID pH 8 saline‐induced gastric contractions were completely eliminated (Fig. 1Bab). On the other hand, treatment with vehicle had no effect on the contractions (data not shown).

In the truncal‐vagotomized animals, the motilin (300 ng/kg)‐induced gastric phase III‐like contractions were the same as those observed in the vagal‐intact suncus (Fig. 1Ca). Similarly, the ID injection of 2 mL of saline with various pH values (pH 6, 7, and 8) resulted in pH‐dependent gastric contractions (Fig. 1Ca). A significant increase in MI was observed, with a maximum achieved with pH 8 saline infusion (Fig. 1Cb), and these contractions induced by ID infusion of pH 8 saline were almost completely abolished by MA or DLS treatment (Figure S1), suggesting that duodenal alkalized conditions stimulated motilin release and then evoked phase III‐like contractions.

### Effect of continuous ID infusion of acidic pH (pH 3) saline on gastric contractions

**Figure 2:**
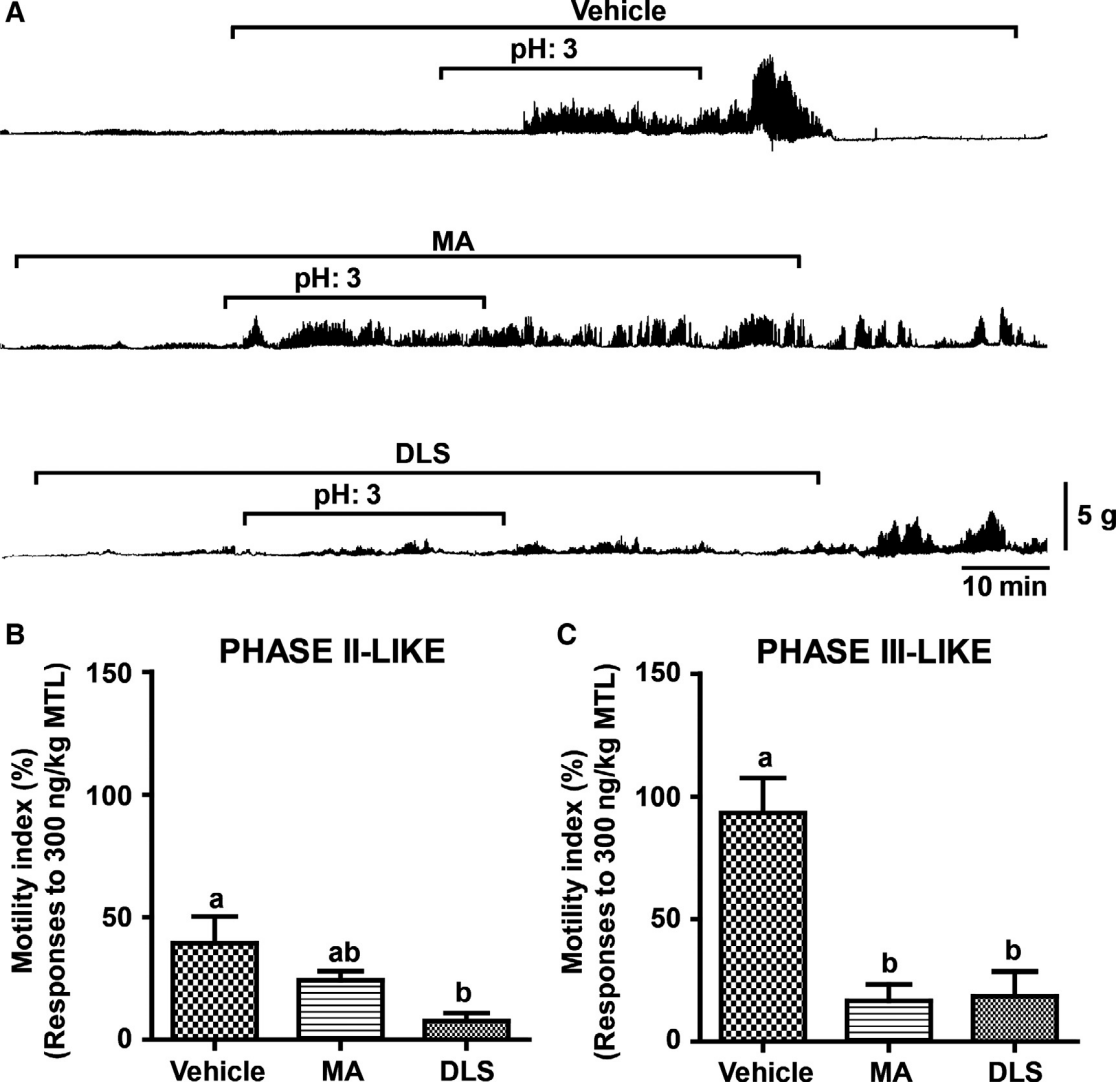
Effect of continuous intraduodenal (ID) infusion of acidic saline on gastric contractions in the anesthetized animal. (A) During and following continuous ID infusion of pH 3 saline (0.1 mL/min, for 30 min) both irregular and strong gastric contractions, respectively, were induced under vehicle treatment. Infusion of MA (1 mg/kg/h, for 1.5 h) or D‐lys3‐GHRP6 (6 mg/kg/h, for 1.5 h) was initiated 20 min prior to continuous infusion of pH 3 saline. (B) The MI of the continuous ID infusion of pH 3 saline‐induced irregular contractions under vehicle, MA, and DLS treatment. A statistically significant difference in MI was observed between vehicle and DLS but not MA. (C) The MI of the pH 3 saline‐induced strong gastric contractions under MA and DLS was significantly decreased from vehicle treatment. Each histogram represents the mean ± standard error of the mean (SEM), *N* = 3; Repeated measures analysis of variance (ANOVA) followed by Tukey's multiple comparison test; Different letters denote significant (*P* < 0.05) differences.

Based on the hypothesis that acidic conditions in the duodenum may change duodenal pH to alkaline conditions through the release of bicarbonate (Flemstrom et al. 1986), we examined whether acidic pH conditions in the duodenum‐ induced gastric contractions. Irregular gastric contractions (phase II‐like) commenced after initiation of the continuous ID infusion of pH 3 saline, which was continued for 30 min, and initiated the strong (phase III‐like) gastric contractions about 10 min after the end of administration (Fig. 2A). Under MA treatment, the ID pH 3 saline‐induced phase II‐like contractions were observed, but no phase III‐like contractions were observed (Fig. 2A). In contrast, both the phase II‐ and III‐like gastric contractions were almost completely eliminated by continuous DLS administration (Fig. 2A). Regarding the phase II‐like contractions, administration of MA and vehicle resulted in no change in the MI, but this was significantly suppressed with DLS treatment (Fig. 2B). On the other hand, the MI of the phase III‐like contractions induced by the ID infusion of pH 3 saline was significantly decreased by the administration of MA or DLS (Fig. 2C).

### Effect of 5HT on inducing gastric contractions

**Figure 3:**
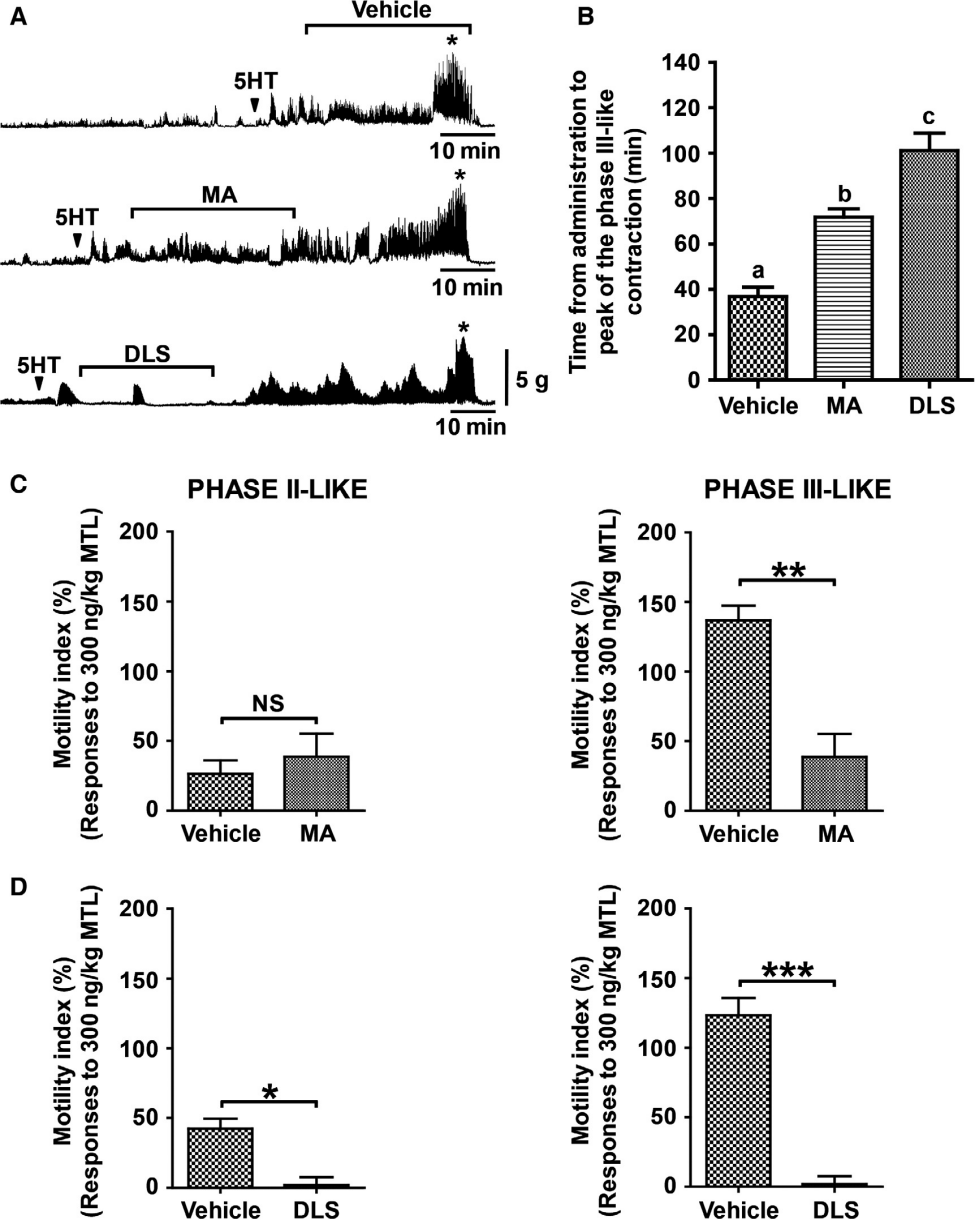
Effect of 5HT on gastric contractions in the anesthetized suncus (A) Typical examples showing that intravenous (IV) bolus infusion of 5HT (10 *μ*g/kg body weight [BW]) induced phase II‐like and phase III‐like gastric contractions. MA or DLS was continuously administered for 30 min at 10–20 min after the injection of 5HT. (B) The duration (min) from the point of administration of 5HT with or without treatment with MA and DLS to the peak of phase III‐like contractions was calculated. Both MA and DLS treatment significantly prolonged the occurrence of phase III‐like contractions. (C) The MI of the 5HT‐induced phase II‐ like contractions did not change with MA treatment, whereas phase III‐like contractions were significantly suppressed. (D) The presence of DLS significantly reduced the MI of both phase II‐ and III‐like contractions induced by the 5HT administration. Each histogram represents the mean ± standard error of the mean (SEM), *N* = 3; Repeated measures analysis of variance (ANOVA) followed by Tukey's multiple comparison test and Student's t‐test (unpaired). *P < 0.05; **P < 0.01; ***P < 0.001. Arrowheads indicate the timing of administration of reagents. Star marks indicate the peak of the 5HT‐induced gastric phase III‐like contractions.

Next, we examined the ability of 5HT to induce gastric contractions in anesthetized suncus. The IV administration of 5HT (10 *μ*g/kg) induced phase II‐ and III‐like gastric contractions (Fig. 3A). The durations of the phase II‐ and III‐like contractions were 33.1 ± 3.7 and 8.3 ± 0.4 min, respectively. The administration of MA for 30 min during the 5HT‐induced phase II‐like contractions did not suppress the phase II‐like contractions but delayed the occurrence of phase III‐like contractions (Fig. 3A). In contrast, DLS treatment abolished the phase II‐like contractions and also prolonged the occurrence of phase III‐like contractions (Fig. 3A). Figure 3B shows that the duration of the occurrence of the peak of the phase III‐like contractions induced by 5HT under vehicle treatment (36.9 ± 4.1 min) were significantly delayed with either MA (71.8 ± 3.6 min) or DLS (101.2 ± 7.6 min) treatment. The MI of the 5HT‐induced gastric phase II‐like contractions remained unchanged during MA treatment, but the phase III‐like contractions were significantly decreased (Fig. 3C). On the other hand, the MI during DLS treatment showed significant suppression of the 5HT‐induced gastric phase II‐ and III‐like contractions (Fig. 3D).

### Involvement of the 5HT4 or 5HT3 receptor on ID pH 3 saline‐induced gastric contractions

**Figure 4:**
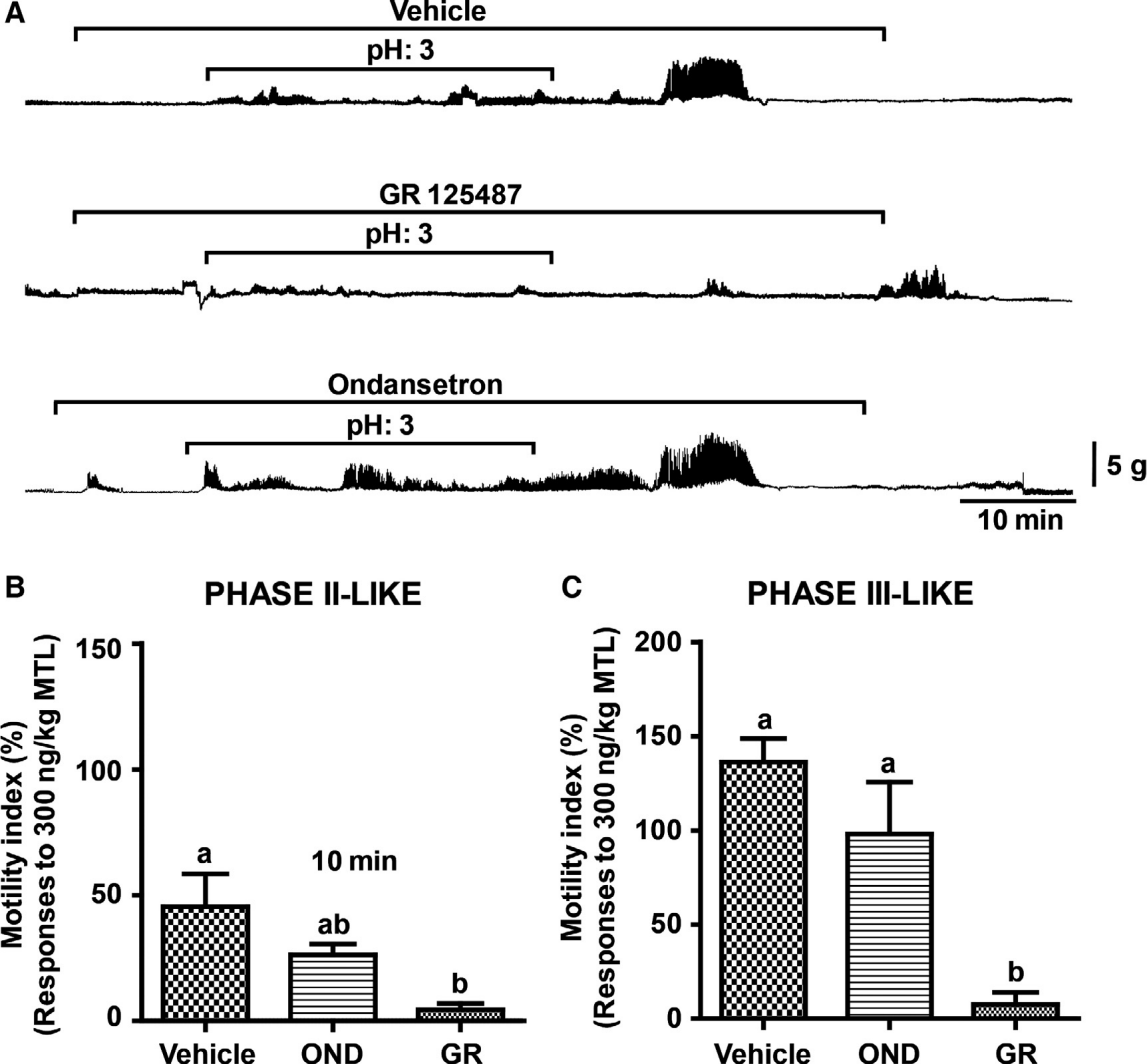
Effect of serotonergic receptor antagonist on the continuous intraduodenal (ID) infusion of pH 3 saline‐induced gastric contractions in the anesthetized condition. (A) Responses of continuous ID infusion of pH 3 saline‐induced phase II‐like irregular contraction and phase III‐like strong contraction under administration of vehicle and GR (80 *μ*g/kg/h, for 1.5 h) or OND (100 *μ*g/kg/h, for 1.5 h). (B and C) The MI of both the phase II‐ and III‐contractions was significantly eliminated under the GR treatment; however, OND failed to suppress the contractions. Each histogram represents the mean ± standard error of the mean (SEM), *N* = 3; Repeated measures analysis of variance (ANOVA) followed by Tukey's multiple comparison test; Different letters denote significant (*P* < 0.05) differences.

We noted characteristics of 5HT‐induced and ID infusion of pH 3 saline‐induced gastric phase II‐ and III‐like contractions. Therefore, we further examined the effect of specific 5HT receptor antagonists on the ID pH 3 saline‐induced gastric contractions to identify the specific receptor involvement in this underlying mechanism. Pretreatment with GR, the selective 5HT4 receptor antagonist, almost completely abolished the phase II‐ and III‐like gastric contractions (Fig. 4A). On the other hand, OND, the 5HT3 receptor antagonist, did not produce any suppressive effect on the phase II‐ and III‐like contractions (Fig. 4A). Insignificant changes in MI of the phase II‐like (Fig. 4B) and phase III‐like (Fig. 4C) gastric contractions were observed between the OND‐ and vehicle‐treated groups. Meanwhile, GR administration significantly decreased the MI of the phase II‐ and III‐like contractions compared to the vehicle‐ or OND‐treated groups (Fig. 4B and C). These results suggest that pH 3 saline‐induced phase II‐ and phase III‐like contractions are mediated by the 5HT4 receptor but not the 5HT3 receptor.

### Effect of 5HT4 receptor agonist on gastric contractions in anesthetized and freely moving fasted animals

**Figure 5:**
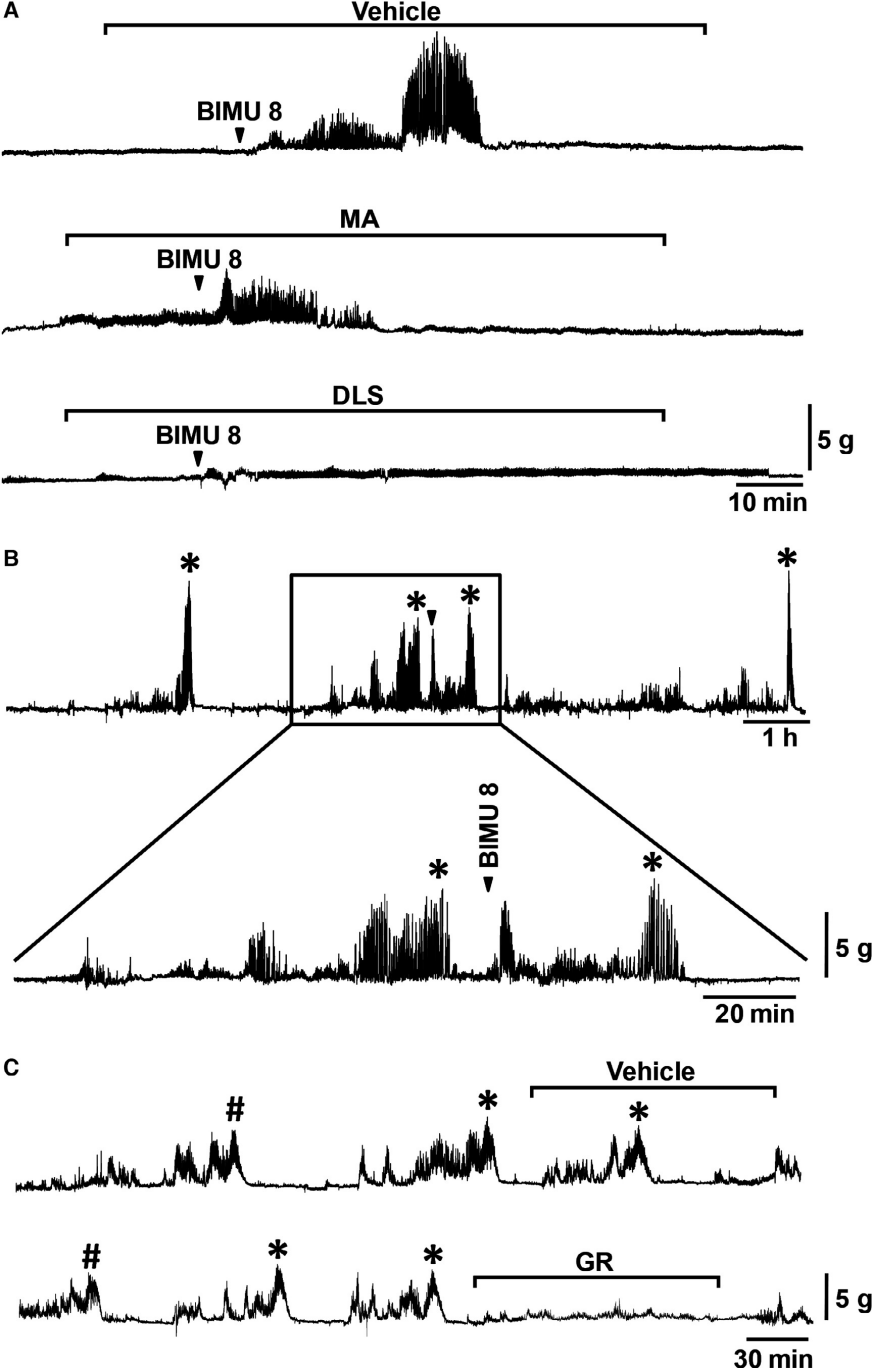
Effect of 5HT4 receptor agonist on gastric contraction and of GR during migrating motor complex (MMC) phase II and phase III contractions. (A) Typical effect of intravenous (IV) bolus administration of BIMU8 (1 mg/kg) on wave pattern of gastric contraction in the anesthetized suncus under administration of vehicle or MA‐2029 (1 mg/kg/h, for 90 min) or DLS (6 mg/kg/h, for 90 min). *N* = 5. (B) Effect of BIMU8 on gastric contraction in the conscious animal. BIMU8 was injected at 10 min of the duration of the end of phase III contractions. *N* = 6. (C) GR 125487 (80 *μ*g/kg/h) was administered IV starting at 70% of the duration of recent phase I and was continued for 2 h. *N* = 2; Arrowheads indicate the timing of administration of reagents. Asterisks indicate spontaneous phase III contractions. #: Postprandial giant contraction (PPGC).

To confirm the role of the 5HT4 receptor in gastric contractions induced by acidic or alkaline pH saline, we examined the effect of BIMU8, a selective 5HT4 receptor agonist, on gastric contractions in the anesthetized suncus. IV bolus administration of BIMU8 (1 mg/kg) evoked irregular (phase II‐like) and strong (phase III‐like) gastric contractions under vehicle treatment (Fig. 5A). Pretreatment with MA suppressed the occurrence of phase III‐like, but not phase II‐like, gastric contractions (Fig. 5A), while administration of DLS completely abolished both the phase II‐ and III‐like contractions (Fig. 5A). A similar result was observed in MI analysis. The MI showed that the MA treatment significantly decreased and almost completely eliminated the phase III‐like, but not phase II‐like, gastric contractions induced by BIMU8 under vehicle treatment (Fig. S2Aab). Conversely, the MI of both the phase II‐ and III‐like contractions was significantly suppressed with IV DLS injection (Figure S2Bab).

We also examined whether BIMU8 could also induce gastric contractions in the freely moving fasted conscious animals. IV bolus administration of BIMU8 (1 mg/kg) during spontaneous phase I contraction induced similar phase II‐ and III‐like contractions (Fig. 5B). We observed the effect of BIMU8 on gastric contractions in six individual animals, and five animals among them showed clear responses. Although we did not observe any gastric contractions following BIMU8 treatment in one animal, this might be due to the animal's incomplete postsurgical recovery, leading to poor body condition and irregular spontaneous MMC (data not shown). To obtain clearer understanding, we further studied the effect of continuous infusion of GR on the spontaneous phase II and III gastric contractions of the MMC. The continuous infusion of GR significantly eliminated the spontaneous contractions in the conscious fasted animal (Fig. 5C).

### Effect of BIMU8 on gastroduodenal contractions in the anesthetized suncus

**Figure 6:**
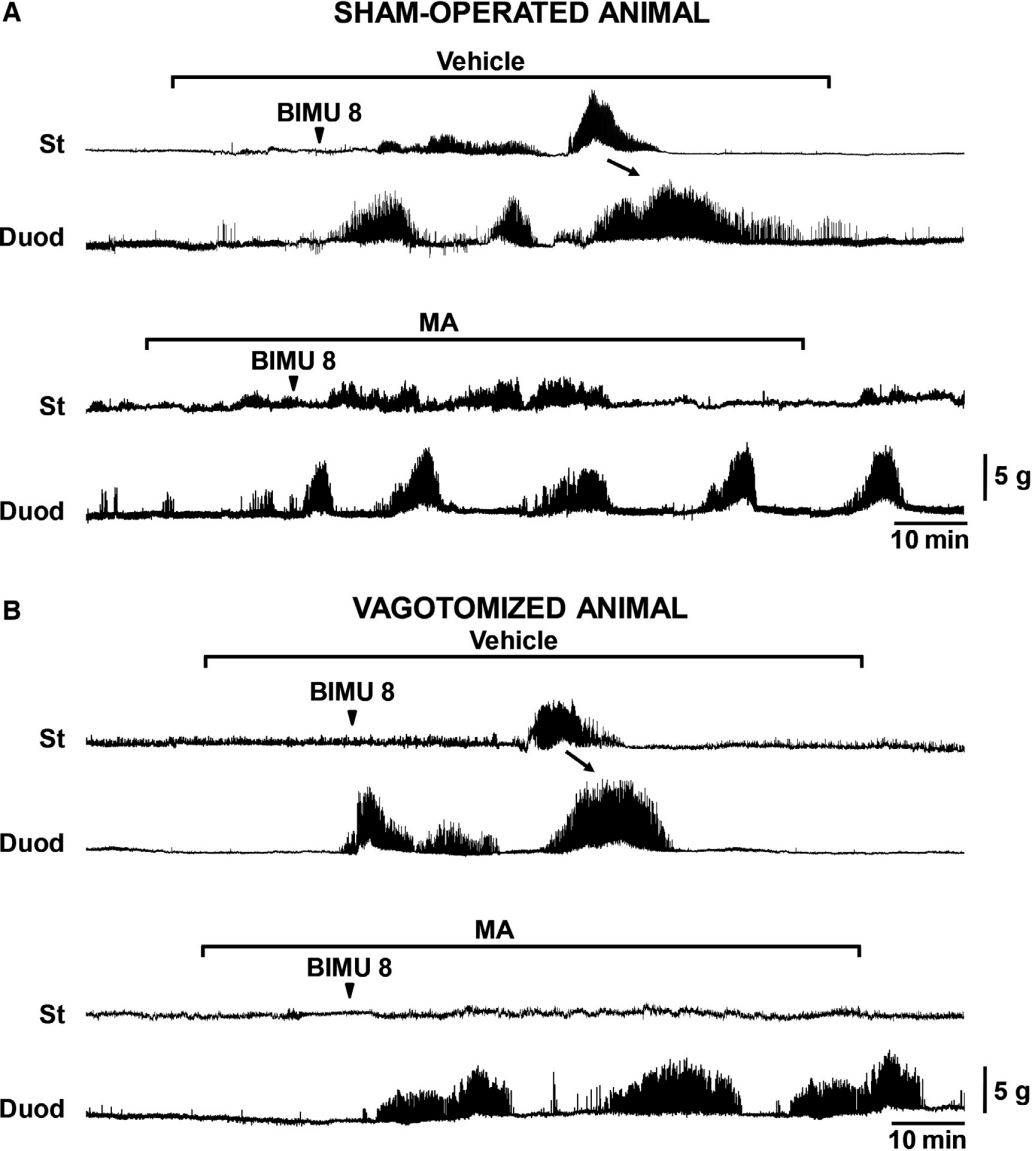
Effect of vagotomy on BIMU8‐induced gastric and duodenal contractions in the anesthetized suncus. (A) Example traces of BIMU8‐induced gastric and duodenal contractions in the sham‐operated suncus. Phase II‐ and III‐like contraction was observed in the stomachs of sham‐operated animals. In the duodenum, shortly after BIMU8 (1 mg/kg) administration, strong and clustered intestinal phase II‐ and III‐like contractions were observed. Continuous MA (1 mg/kg/h, for 1.5 h) eliminated the occurrence of gastric phase III‐like, but not phase II‐like and duodenal contractions. (B) BIMU8‐induced gastric and duodenal contractions in the vagotomized animal. BIMU8‐induced gastric phase II‐like contraction was absent, although duodenal contraction was unaffected by vagotomy. MA‐2029 treatment demonstrated that BIMU8‐induced gastric phase III‐like contraction was eliminated but duodenal contractions were not affected. *N* = 3. Arrows indicate the propagated gastric phase III‐like contraction to duodenum. Arrowheads indicate the timing of administration of reagents. St: Stomach; Duod: Duodenum.

It has already been reported that the vagal nerve is important for spontaneous phase II contraction, although it does not play a role in phase III contraction (Miyano et al. 2013). Moreover, the involvement of the 5HT4 receptor in the duodenal MMC was observed in a study on dogs (Nakajima et al. 2010). Therefore, we examined the effect of BIMU8 on gastroduodenal contractions and compared the effect between sham‐operated and vagotomized animals. In the sham‐operated suncus, the phase II‐like gastric contractions were unaffected by MA and vehicle treatment, although phase III‐like gastric contractions were suppressed under MA infusion (Fig. 6A and Fig. S3A). In contrast, in the vagotomized animal, no BIMU8‐induced gastric phase II‐like contractions were visible, but phase III‐like contractions were clearly visible (Fig. 6B and Fig. S3A), and significantly diminished by MA treatment (Fig. 6B and Fig. S3A).

In the duodenum, bolus IV administration of BIMU8 dramatically induced clustered and continuous contraction and ended with the propagated contraction of gastric phase III‐like contractions known as intestinal phase II‐ and III‐like contractions (Fig. 6A and B), as observed in the interdigestive phase II and III contractions of the small intestine in the conscious state (manuscript under preparation). The representative wave response figure (Fig. 6A and B) and MI (Figure S3B) showed that neither the phase II‐like nor the phase III‐like duodenal contractions induced by BIMU8 were affected by vagotomy and/or MA treatment.

### Effect of indomethacin on gastroduodenal contractions induced by ID infusion of pH 3 saline or BIMU8

**Figure 7:**
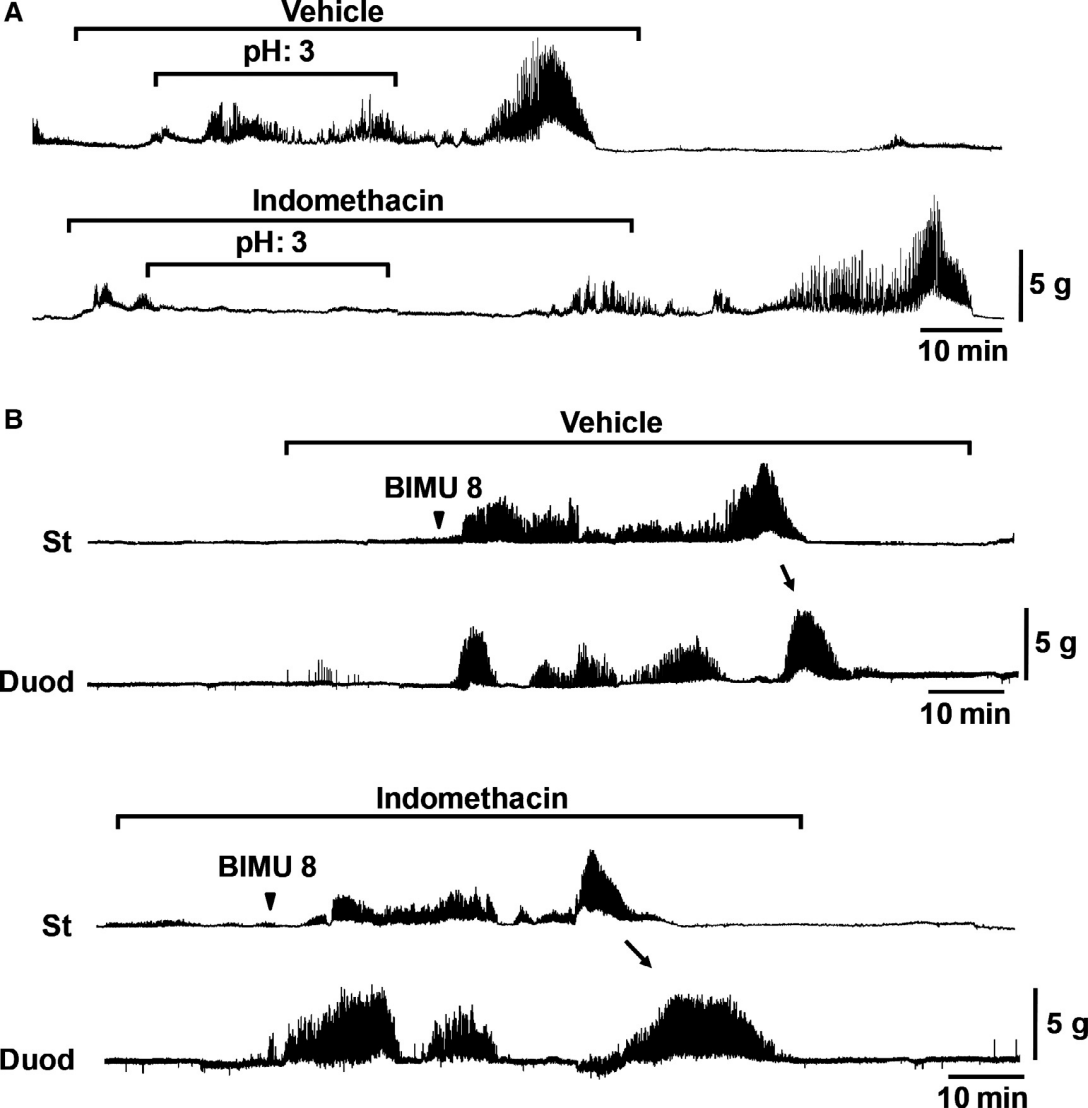
Effect of indomethacin on continuous intraduodenal (ID) infusion of pH 3 saline‐induced gastric contraction and BIMU8‐induced gastric and duodenal contractions in the anesthetized suncus. (A) Vehicle or Indo (10 mg/kg/h) was continuously intravenously (IV) administered for 70 min. The ID infusion of pH 3 saline (0.1 mL/min for 30 min) was initiated at 10 min after administration of Indo or vehicle. pH 3‐induced contractions were eliminated by Indo treatment. (B) Example of BIMU8‐induced gastric and duodenal contractions under the continuous infusion of Indo (10 mg/kg/h, for 1.5 h) or vehicle. Indo had no effect on either gastric or duodenal contractions evoked by BIMU8. *N* = 3. Arrowhead indicates the timing of administration of reagents. Arrows indicate the propagated gastric phase III‐like contraction to duodenum. St: Stomach; Duod: Duodenum.

Many studies have shown that prostaglandin (PG) is also involved in duodenal acid‐stimulated bicarbonate secretion. Therefore, we speculated that PG would play an important role in the regulation of duodenal pH changes to induce gastric contractions. We observed that the continuous infusion of Indo, the prostaglandin E_2_ (PGE_2_) inhibitor, abolished both the phase II‐ and III‐like contractions induced by ID infusion of pH 3 saline (Fig 7A and Fig. S4). On the other hand, the BIMU8‐induced gastroduodenal contractions were not inhibited in the presence of vehicle or Indo (Fig. 7B and Fig. S5). These results indicate that duodenal low pH triggers the series of MMC events, and Indo release is positioned upstream of 5HT release.

## Discussion

Brown et al. (1966) demonstrated in 1966 that an alkaline pH in the duodenum increases gastric motility, suggesting that this occurs through the release of some humoral stimulating agent. Itoh et al. (1980) found that in dogs, fasting irregular contractions are associated with lower duodenal pH and there is a dramatic increase to alkaline pH during strong regular contractions. An almost identical observation was reported in a human study, which found that duodenal pH fluctuates from 2 to 7.5 during the onset of phase II, whereas the pH remains alkaline from late phase II to phase III contractions (Woodtli and Owyang 1995). Moreover, results in dogs showed increased gastric motor activity in association with increased serum motilin concentration soon after the duodenal alkalization (Dryburgh and Brown 1975). Therefore, it is conceivable to hypothesize that during the interdigestive state, duodenal alkaline pH might have an influential effect on motilin release, as the plasma motilin peak is associated with the occurrence of gastric phase III in humans (Vantrappen et al. 1979a; Janssens et al. 1983) and dogs (Itoh et al. 1976, 1978; Hall et al. 1983). In accordance with these results, we also observed duodenal pH 8‐induced strong phase III‐like gastric contractions in the suncus, even in the vagotomized condition. Although motilin is considered the main stimulator of phase III contractions of the MMC, it has already been reported that a significant amount of ghrelin is also essential to initiate the contractions (Kuroda et al. 2015). Correspondingly, we also observed that gastric contractions induced by the ID infusion of pH 8 saline were considerably decreased by MA or DLS treatment, suggesting that the duodenal alkalinized condition may be important for the endogenous release of motilin into the bloodstream and the induction of gastric contractions in coordination with ghrelin. These results together support the further assumption that duodenal alkaline pH is the main signal for intestinal motilin release to regulate gastric phase III contractions.

The association between the cyclic release of gastric acid and phase II activity has already been reported (Vantrappen et al. 1979b). Moreover, several reports have demonstrated that small intestinal luminal acidification stimulates the release of motilin and controls the MMC. For example, the proximal part of the duodenum is considered the most responsive site of motilin release under acidic pH conditions in a physiological manner in the pig (Cuber et al. 1985). A previous human study showed that duodenal acidification increased motilin release by 90% (Mitznegg et al. 1976), but a dog study showed that motilin release was independent from duodenal acidification (Dryburgh and Brown 1975). Nevertheless, these studies did not clarify how duodenal acidification stimulates motilin release. Considering these results, we further supposed that there is a continuous flow of gastric acid to the proximal part of the intestine, and then across the threshold to result in the secretion of bicarbonate to create an alkaline pH condition, which finally stimulates the release of motilin regulating gastric phase III contractions of the MMC in the suncus. This assumption is supported by the present result that characteristic phase II‐ like gastric contractions appeared during continuous ID infusion of pH 3 saline, and phase III‐like gastric contractions appeared after the end of the administration. Moreover, the MI also showed a similar suppressive MI pattern under MA and DLS treatment as in the case of spontaneous MMC in the suncus (Mondal et al. 2012). Therefore, it may be speculated that the rhythmic changes in duodenal pH may be one of the important regulators of the gastric MMC, where intestinal acidic pH could be an initiator and alkaline pH is the final modulator of motilin release controlling MMC cyclically within a regular interval during fasting conditions.

A relationship between 5HT and GI motility has been reported in many studies. Administration of 5HT in dogs stimulates intestinal phase II‐like contractions (Ormsbee et al. 1984) followed by gastric phase III‐contractions in accordance with the increase in the plasma motilin level (Nakajima et al. 2010). In line with these results, we found that IV administration of 5HT clearly induced gastric phase II‐ and III‐like contractions. Furthermore, MA and DLS significantly delayed the occurrence of 5HT‐induced gastric phase III‐like contractions. These results indicate that the 5HT‐induced gastric contraction is also mediated by the endogenous release of motilin in association with ghrelin. The above results show that an identical mechanism exists with the gastric contractions induced by ID infusion of pH 3 saline. 5HT has been identified as one of the important molecules involved in the secretory response to duodenal luminal acid (Flemstrom et al. 1986; Allen and Flemstrom 2005) and raises the pH to alkaline by luminal bicarbonate secretion (Tuo and Isenberg 2003; Tuo et al. 2004; Safsten et al. 2006). Therefore, it is conceivable that, in the present study, 5HT could be regulating the gastric MMC indirectly through coordinating the acid‐stimulated bicarbonate release to stimulate motilin. Therefore, we further examined whether ID pH 3 saline‐induced gastric contractions were suppressed by 5HT receptor antagonist treatment. Interestingly, we observed that GR (the 5HT4 receptor antagonist), but not OND (the 5HT3 receptor antagonist), completely eliminated the ID pH 3 saline‐induced gastric phase II‐ and III‐like contractions. Reports in studies of rats and mice demonstrated that the 5HT4, but not the 5HT3 receptor (Safsten et al. 2006), is a component of the regulatory pathway for the 5HT‐stimulated duodenal mucosal secretion of bicarbonate (Tuo et al. 2004; Safsten et al. 2006). These observations again convinced us to speculate that duodenal acid may regulate 5HT release, and, through 5HT4 receptor stimulation, result in secretion of bicarbonate into the duodenum and increase in the duodenal pH to alkaline conditions, leading to the release of motilin for maintenance of the MMC.

In studies in dogs, it has been suggested that an interaction between 5HT and motilin with a positive feedback mechanism mediates the MMC cycle (Nakajima et al. 2010). It has also been demonstrated that the 5HT3 receptor is involved in gastric phase III via the vago‐vagal reflex (Itoh et al. 1991; Nakajima et al. 2010), while the 5HT4 receptor regulates both gastric and intestinal MMC (Nakajima et al. 2010). Although the involvement of the vagus nerve in the gastric spontaneous phase III contractions remains controversial (Hall et al. 1983; Lemoyne et al. 1984; Yoshiya et al. 1985; Tanaka et al. 2001), it has already been shown that the vagus nerve is not involved in the regulation of phase III contractions of the MMC in the study of suncus (Miyano et al. 2013). Thus, in suncus, the 5HT4 receptor alone may be the important regulator of the GI MMC. To support this hypothesis, we next clearly demonstrated that IV administration of BIMU8, the selective 5HT4 receptor agonist, also induced similar characteristically phase II‐ and phase III‐like gastric contractions. Accordingly, MA treatment only suppressed the BIMU8‐induced phase III‐like contractions, while DLS significantly eliminated both phase II‐ and III‐like gastric contractions in the anesthetized suncus. To verify this result in the conscious suncus, we observed that IV BIMU8 injection also clearly induced phase II‐ and III‐like gastric contractions that were comparable to spontaneous MMC in the fasted condition. Moreover, as expected, prolonged GR treatment in the fasted conscious animal almost completely eliminated the spontaneous phase II and III gastric contractions of the MMC in the present study. These results together indicated that 5HT4 could be the main intermediate molecule to regulate duodenal changes in pH controlling the gastric MMC by release of motilin. However, how and whether the gastric phase II and the 5HT4 receptor are involved in intestinal MMC remains to be elucidated.

To answer these questions, we further studied the effect of BIMU8 on the gastroduodenal contractions in the sham‐operated and vagotomized suncus. No phase II‐like contractions were observed with 5HT4 agonist treatment in the vagotomized suncus. In contrast, 5HT4 agonist treatment clearly induced clustered duodenal phase II‐ and III‐like contractions in the vagotomized animals or under MA treatment. These results further suggest that 5HT4 may also be responsible for stimulating the vago‐vagal reflex to induce gastric phase II contractions, whereas propagated duodenal MMC showed vagus‐independent contractions. In addition, the gastric phase III contractions induced by the 5HT4 receptor are thought to arise in an indirect manner through increases in duodenal pH to alkaline, leading to motilin release. Measuring duodenal bicarbonate and/or pH under conditions of 5HT4 agonist treatment could verify our assumption. To explain the mechanism of the spontaneous gastric phase II contractions using suncus, it was previously speculated that ghrelin is responsible (Mondal et al. 2012) through the vago‐vagal pathway (Miyano et al. 2013). However, our recent results demonstrated that a certain amount of ghrelin acts as an effector molecule for motilin‐induced gastric contraction. Moreover, we think that 5HT, through the 5HT4 receptor, initiates the occurrence of gastric phase II contractions via the vago‐vagal reflex. These preliminary findings will require confirmation through direct evidence and verification of the morphological existence of the 5HT4 receptor in the vagal pathway.

We also found that prolonged treatment with Indo significantly suppressed the ID pH 3 saline‐induced phase II‐ and III‐like contractions, suggesting the probable involvement of PGs in the duodenal changes in pH for controlling the MMC. PGs are widely distributed in the GI tissue and associated with numerous physiological activities. For example, the acid‐induced duodenal bicarbonate secretion was stimulated through PGE_2_ (Hirokawa et al. 2004), PGE_2_ also plays a role in regulation of intestinal MMC (Mochiki et al. 2000), and suppress the release of gastric pepsin and acid (Mihas et al. 1976). Mochiki et al. (2000) also reported in a study on dogs that PGE_2_‐*α* may partly stimulate the cyclic release of motilin. However, we consider that PGE_2_‐*α* may be indirectly involved and may interact with the 5HT4 receptor to regulate the duodenal changes of pH for the release of motilin, as its inhibitor and antagonist inhibit the gastric contractions induced by the ID infusion of pH 3 saline. We also observed that Indo treatment did not eliminate either stomach or duodenal contractions induced by 5HT4 agonist administration. This result further indicates that the duodenal acid may generate the release of PGE_2_‐*α*, which might stimulate 5HT release and result in luminal bicarbonate secretion into the intestine through the 5HT4 receptor. As both the PGE_2_ and 5HT4 receptors facilitate cholinergic neurotransmission in the myenteric plexus (Kilbinger and Wolf 1992; Mulholland and Simeone 1993), there may be the possibility that PGE_2_ facilitates 5HT release through stimulation of the 5HT4 receptor via the myenteric plexus.

**Figure 8:**
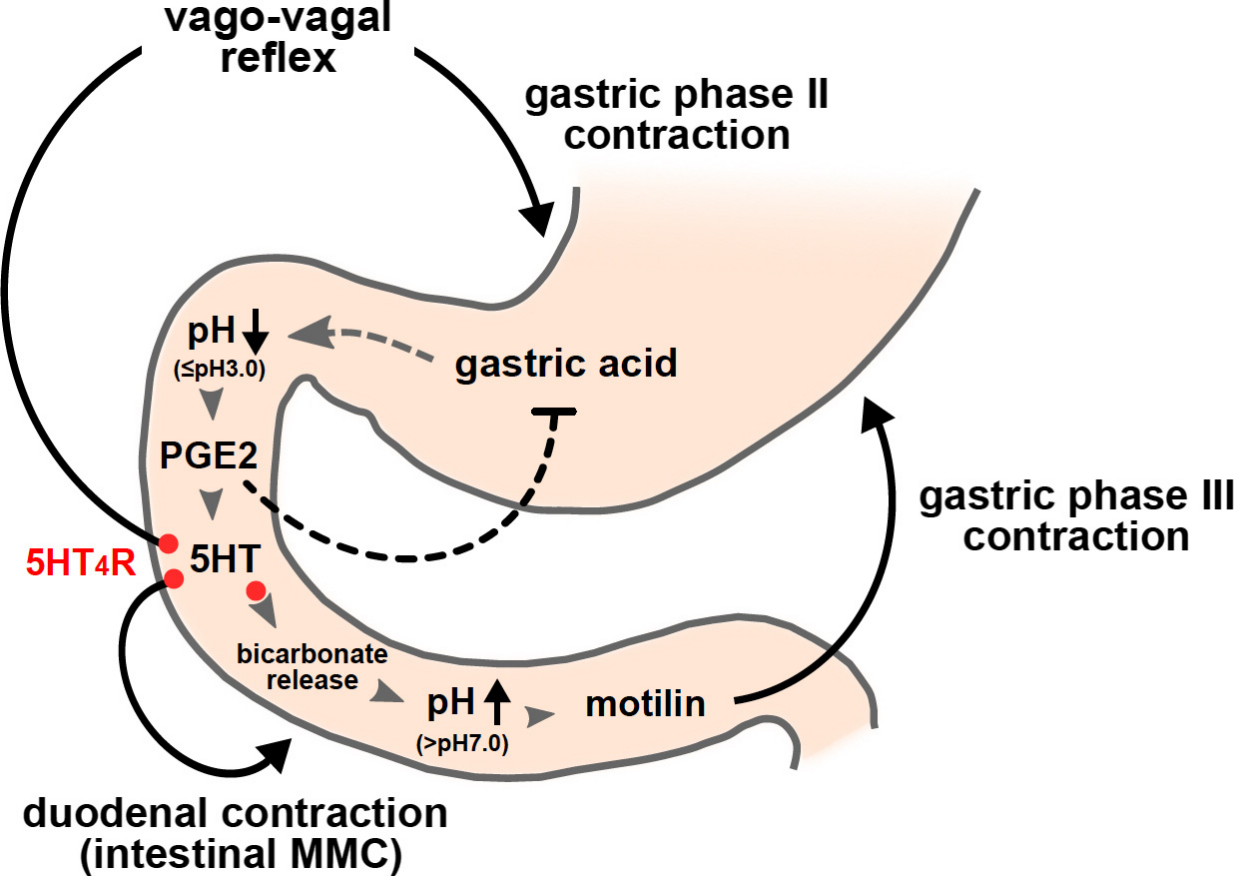
Proposed mechanism regulating gastric phase III contractions of the migrating motor complex (MMC) in suncus**.**

The ultradian rhythm of gastric acidity in a 90‐min period has been recorded in rhesus monkeys (Hamilton and Natelson 1984). Moreover, the clear ultradian rhythmic pattern of the occurrence of the MMC (90–120 min period) in humans, dogs, and suncus has already been demonstrated (Hiatt and Kripke 1975; Sakahara et al. 2010; Takahashi 2012). Based on these demonstrations and together with our results, we would like to propose that the GI MMC might be mediated through the change in duodenal pH. Our predicted hypothesis for the regulation of the GI MMC is demonstrated in Figure 8. Briefly, before the onset of gastric phase II contractions, released gastric acid flows down to the duodenum, and this triggers the release of PGE_2_ to stimulate 5HT release. 5HT initiates the vago‐vagal reflex regulating phase II contractions together with duodenal phase II contractions through the 5HT4 receptor. Moreover, 5HT increases duodenal pH by luminal bicarbonate secretion, while PGE_2_ may play a role in stopping gastric acid, leading to changes to alkaline pH to release motilin. In this way, gastric phase III of the MMC may be regulated in *S. murinus*. The present study, however, did not identify the possible mechanism for motilin release due to the duodenal alkalization. Nevertheless, our study proposes that the continuous flow of the gastric acid to the small intestine would be the originator of and would subsequently control the interdigestive MMC through changes in duodenal pH to alkali condition and the endogenous release of motilin.

## Conclusion

The physiological importance of the cyclic pH changes seems very likely to be related to the cleansing and eradication of bacterial overgrowth in the GI tract during the interdigestive state (Vantrappen et al. 1977). Moreover, whether the impaired phase III activity in patients with functional dyspepsia may be mediated by abnormal fluctuation of GI pH needs to be investigated. Thus, the results of the present study offer novel insights regarding the mechanism of GI acid and bicarbonate secretion in relation to future perspectives on the treatment of disorders of impaired motility or other related diseases.

## Conflict of Interest

Authors do not have any competing interests.

## Supporting information




**Figure S1.** Effect of MA 2029 and D‐lys3‐GHRP6 on the intraduodenal (ID) infusion of pH 8 salineinduced gastric contraction in the vagotomized suncus.

**Figure S2.** The motility index (MI) of the effect of MA‐2029 and D‐lys3‐GHRP6 on BIMU8‐induced gastric contraction in the anesthetized suncus.

**Figure S3.** The MI of the effect of BIMU8 on gastric and duodenal contraction in the sham‐operated and vagotomized suncus (A) MI calculated in the phase II‐ and III‐like gastric contraction induced by BIMU8 administration.

**Figure S4.** The MI of the effect of indomethacin (Indo) on the gastric contractions induced during and after the continuous intraduodenal (ID) administration of pH 3 saline (A) MI of the ID infusion of pH 3 saline‐induced gastric phase II‐like contractions in the presence of vehicle or Indo.

**Figure S5.** The MI of the effect of indomethacin (Indo) on the BIMU8‐induced gastric and duodenal contractions (A) MI of the phase II‐ and III‐like gastric contractions induced by BIMU8 treatment under Indo infusion was observed.
