# Supplementary material for: Underlying mechanism of the cyclic migrating motor complex in Suncus murinus: a change in gastrointestinal pH is the key regulator
Source: Physiol Rep. 2017 Jan 13;5(1):e13105. doi: 10.14814/phy2.13105 (PMC5256163; doi:10.14814/phy2.13105)
Supplement: Supplementary file 1 — Figure S1. Effect of MA 2029 and D‐lys3‐GHRP6 on the intraduodenal (ID) infusion of pH 8 salineinduced gastric contraction in the vagotomized suncus. [file PHY2-5-e13105-s001.pdf]

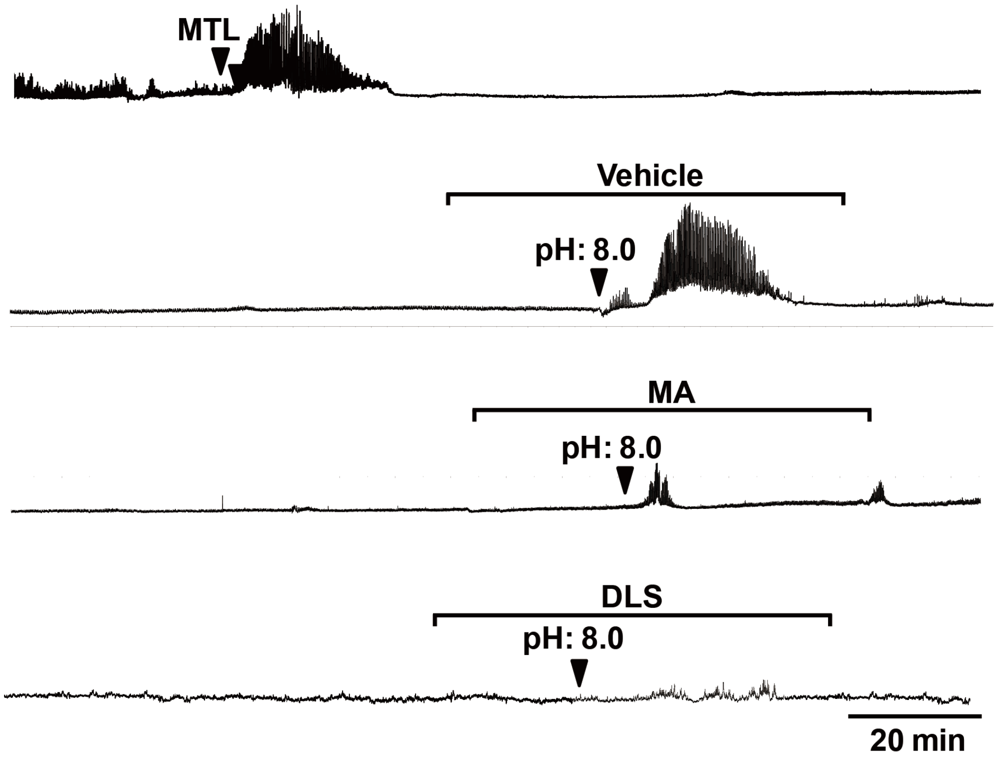

Supplementary Figure 1

Effect of MA 2029 and D-lys3-GHRP6 on the intraduodenal (ID) infusion of pH 8 saline-induced gastric contraction in the vagotomized suncus. Representative traces showing the control contraction induced by motilin (300 ng/kg BW) and the ID infusion of pH 8 saline-induced contractions. Either MA-2029 (MA; 1 mg/kg/h, for 1 h) or D-lys3-GHRP6 (DLS; 6 mg/kg/h, for 1 h) infusion was started 20 min before the ID infusion of pH 8 saline. ID infusion of both MA and DLS eliminated alkaline saline-evoked gastric contraction. N = 3. Arrowheads indicate the timing of administration of reagents.
