# Supplementary material for: Underlying mechanism of the cyclic migrating motor complex in Suncus murinus: a change in gastrointestinal pH is the key regulator
Source: Physiol Rep. 2017 Jan 13;5(1):e13105. doi: 10.14814/phy2.13105 (PMC5256163; doi:10.14814/phy2.13105)
Supplement: Supplementary file 2 — Figure S2. The motility index (MI) of the effect of MA‐2029 and D‐lys3‐GHRP6 on BIMU8‐induced gastric contraction in the anesthetized suncus. [file PHY2-5-e13105-s002.pdf]

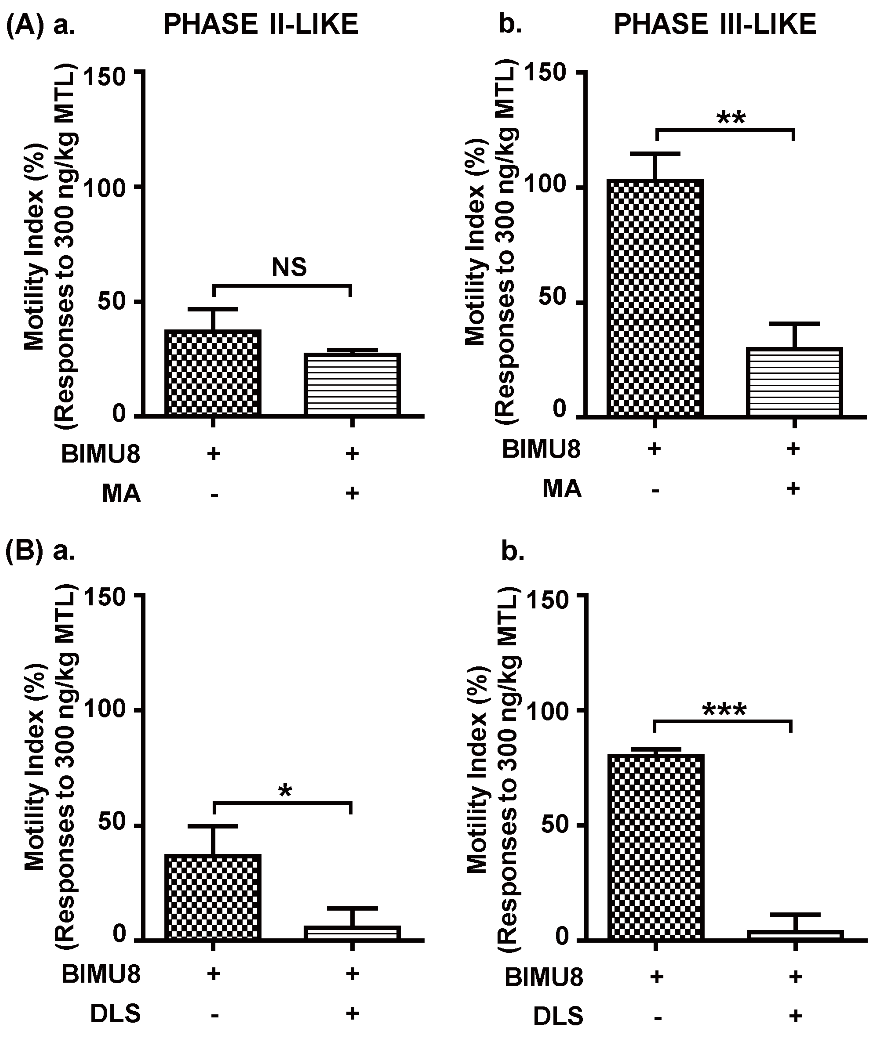

Supplementary Figure 2

The motility index (MI) of the effect of MA-2029 and D-lys3-GHRP6 on BIMU8-induced gastric contraction in the anesthetized suncus. (A) The MI of the phase II-like (a) and phase III-like (b) gastric contraction in the presence or absence of MA. The MI of the phase III-like, but not phase II-like, contraction was significantly decreased in the presence of MA. (B) The MI of BIMU8-induced phase II-like (a) and phase III-like (b) gastric contraction was almost completely eliminated by DLS administration. The methodology followed to calculate the MI is described in the Materials and Methods section. Mean ± SEM. Student's t test; N = 5, * $P < 0.05$; ** $P < 0.01$; *** $P < 0.001$; NS, $P > 0.05$.
