# Supplementary material for: Underlying mechanism of the cyclic migrating motor complex in Suncus murinus: a change in gastrointestinal pH is the key regulator
Source: Physiol Rep. 2017 Jan 13;5(1):e13105. doi: 10.14814/phy2.13105 (PMC5256163; doi:10.14814/phy2.13105)
Supplement: Supplementary file 3 — Figure S3. The MI of the effect of BIMU8 on gastric and duodenal contraction in the sham‐operated and vagotomized suncus (A) MI calculated in the phase II‐ and III‐like gastric contraction induced by BIMU8 administration. [file PHY2-5-e13105-s003.pdf]

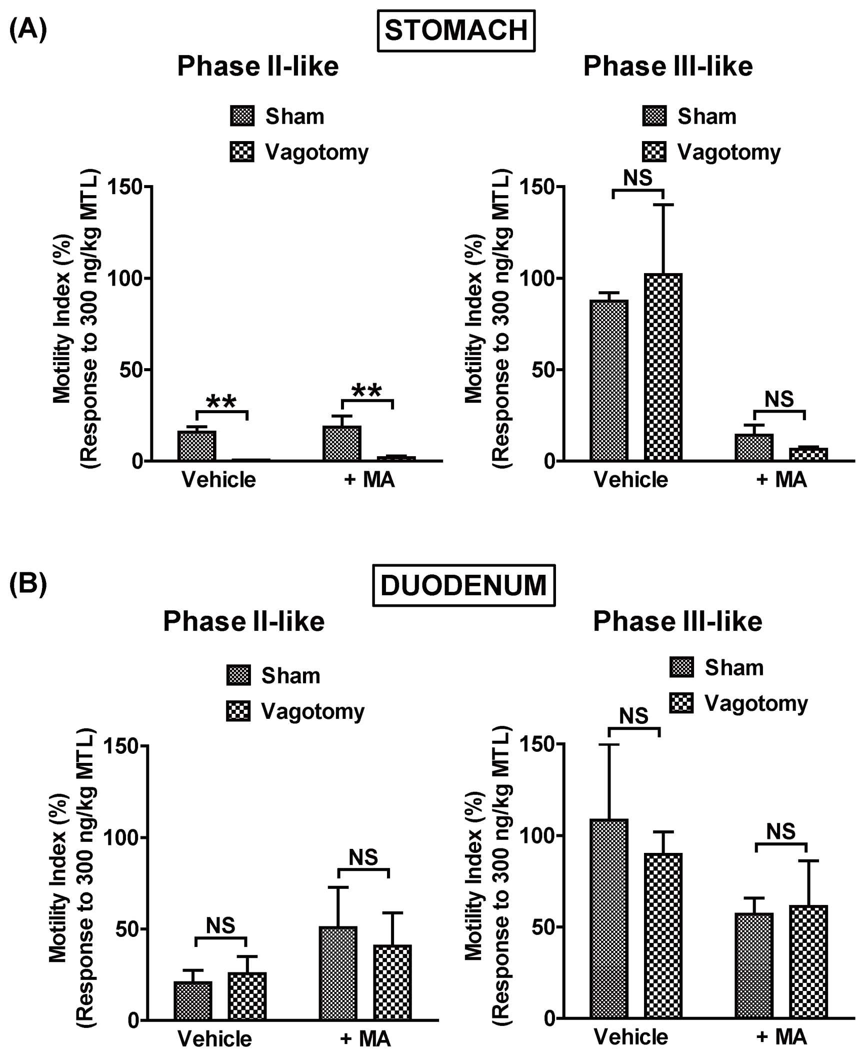

Supplementary Figure 3

The MI of the effect of BIMU8 on gastric and duodenal contraction in the sham-operated and vagotomized suncus (A) MI calculated in the phase II- and III-like gastric contraction induced by BIMU8 administration. Vagotomy significantly reduced the MI of the gastric phase-II like contraction. On the other hand, the MI of the gastric phase III-like contraction remained insignificant in the vagotomized animal but decreased with motilin antagonist treatment. (B) MI of the BIMU8-induced duodenal phase II- and III-like contractions in the absence and presence of MA in the sham-operated and vagotomized suncus. Neither vagotomy nor MA had any significant effect on the MI of the duodenal phase II- and III-like contractions. The methodology followed to calculate the MI is described in the Materials and Methods section. Mean ± SEM. Student's t-test (unpaired); **$P < 0.01$; NS, $P > 0.05$; $N = 3$.
