# Supplementary material for: Underlying mechanism of the cyclic migrating motor complex in Suncus murinus: a change in gastrointestinal pH is the key regulator
Source: Physiol Rep. 2017 Jan 13;5(1):e13105. doi: 10.14814/phy2.13105 (PMC5256163; doi:10.14814/phy2.13105)
Supplement: Supplementary file 4 — Figure S4. The MI of the effect of indomethacin (Indo) on the gastric contractions induced during and after the continuous intraduodenal (ID) administration of pH 3 saline (A) MI of the ID infusion of pH 3 saline‐induced gastric phase II‐like contractions in the presence of vehicle or Indo. [file PHY2-5-e13105-s004.pdf]

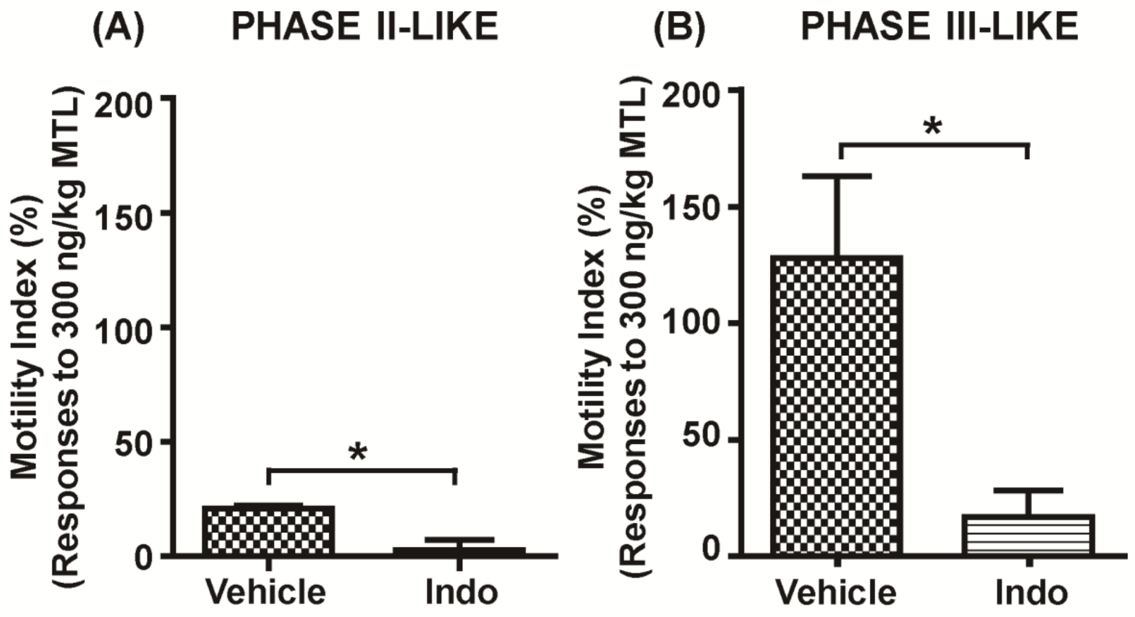

Supplementary Figure 4

The MI of the effect of indomethacin (Indo) on the gastric contractions induced during and after the continuous intraduodenal (ID) administration of pH 3 saline (A) MI of the ID infusion of pH 3 saline-induced gastric phase II-like contractions in the presence of vehicle or Indo. (B) MI of the ID infusion of pH 3 saline-induced gastric phase III-like contractions in the presence of vehicle or Indo. The methodology followed to calculate the MI is described in the Materials and Methods section. Mean ± SEM. Student's t test; * $P < 0.05$; $N = 3$.
