# Supplementary material for: Underlying mechanism of the cyclic migrating motor complex in Suncus murinus: a change in gastrointestinal pH is the key regulator
Source: Physiol Rep. 2017 Jan 13;5(1):e13105. doi: 10.14814/phy2.13105 (PMC5256163; doi:10.14814/phy2.13105)
Supplement: Supplementary file 5 — Figure S5. The MI of the effect of indomethacin (Indo) on the BIMU8‐induced gastric and duodenal contractions (A) MI of the phase II‐ and III‐like gastric contractions induced by BIMU8 treatment under Indo infusion was observed. [file PHY2-5-e13105-s005.pdf]

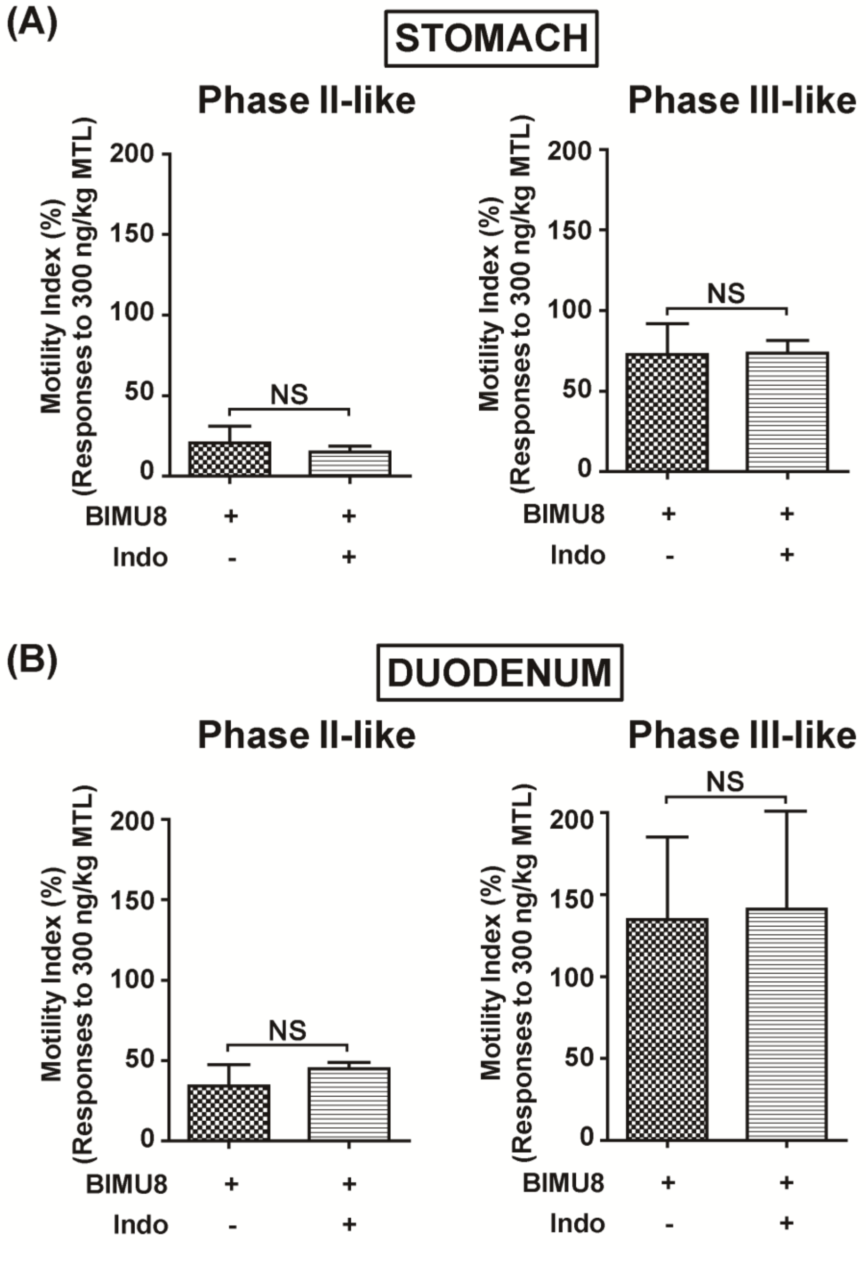

Supplementary Figure 5

The MI of the effect of indomethacin (Indo) on the BIMU8-induced gastric and duodenal contractions (A) MI of the phase II- and III-like gastric contractions induced by BIMU8 treatment under Indo infusion was observed. Neither phase II-like nor phase III-like MI of the gastric contractions was suppressed by Indo treatment. (B) Similar to the stomach contractions, Indo treatment did not show any effect on the MI of the BIMU8-induced phase II- and III-like duodenal contractions. The methodology followed to calculate the MI is described in the Materials and Methods section. Mean ± SEM. Student's t-test (unpaired); NS, $P > 0.05$; $N = 3$.
